# Temperature dependence of the thermo-optic coefficient in 4H-SiC and GaN slabs at the wavelength of 1550 nm

**DOI:** 10.1038/s41598-022-08232-x

**Published:** 2022-03-21

**Authors:** Sandro Rao, Elisa D. Mallemace, Giuseppe Cocorullo, Giuliana Faggio, Giacomo Messina, Francesco G. Della Corte

**Affiliations:** 1 Department DIIES, Mediterranea University, 89124 Reggio Calabria, Italy; 2 Department DIMES, University of Calabria, 87036 Cosenza, Italy; 3 Department DIETI, University of Naples Federico II, 80125 Naples, Italy

**Keywords:** Electrical and electronic engineering, Photonic devices

## Abstract

The refractive index and its variation with temperature, i.e. the thermo-optic coefficient, are basic optical parameters for all those semiconductors that are used in the fabrication of linear and non-linear opto-electronic devices and systems. Recently, 4H single-crystal silicon carbide (4H-SiC) and gallium nitride (GaN) have emerged as excellent building materials for high power and high-temperature electronics, and wide parallel applications in photonics can be consequently forecasted in the near future, in particular in the infrared telecommunication band of λ = 1500–1600 nm. In this paper, the thermo-optic coefficient (*dn/dT*) is experimentally measured in 4H-SiC and GaN substrates, from room temperature to 480 K, at the wavelength of 1550 nm. Specifically, the substrates, forming natural Fabry–Perot etalons, are exploited within a simple hybrid fiber free-space optical interferometric system to take accurate measurements of the transmitted optical power in the said temperature range. It is found that, for both semiconductors, *dn/dT* is itself remarkably temperature-dependent, in particular quadratically for GaN and almost linearly for 4H-SiC.

## Introduction

Over the last decade, we seen the rapid development of industrial applications of microelectronic devices based on new semiconducting materials, such as Silicon Carbide (SiC) and Gallium Nitride (GaN). Power electronic applications, in particular, have taken huge advantages from the adoption of these wide bandgap semiconductors, both of which allow the effective fabrication of high breakdown voltage, low on-resistance, diodes and transistors^1–4^, capable moreover of operating at much higher temperatures compared to Silicon (Si) ones^5,6^. As the deployment of these materials in electronics is destined to grow in the coming years^7^, it can be expected that this will trigger the development of passive and active photonic devices for communication or sensing purposes, possibly monolithically integrated within the same chip^8–10^. An obvious prerequisite for the development of such devices is the precise knowledge of the optical properties of these materials, in particular their dependence on temperature variations that can be reached during the operating life.

However, although the temperature dependence of the refractive index is essential for understanding the optical potential of novel materials, such as 4H-SiC and GaN, investigations on the thermo-optic coefficient (TOC) are limited. To date, only a few studies on these wide-bandgap materials can be found in the literature^11^, based on different experimental methods, such as interferometry^12^, z-scan^13^, thermal lens^14^ and light-induced transient thermal grating techniques^15^, and carried out in various wavelength ranges.

In particular, for the hexagonal (4H) polytype of SiC, although usually regarded among the best materials for high-power electronic and optoelectronic applications^16^, an accurate value of the TOC and its temperature dependence is still lacking at the common fiber-optic wavelengths of λ ~ 1.55 μm.

Scajev et al.^15^ applied a time-resolved four-wave mixing technique for the determination of TOCs in heavily doped n-type and p-type 4H-SiC substrates at room temperature (RT). Spatial modulation of thermal properties was achieved by intraband carrier excitation using a light-interference pattern at λ = 1064 nm and subsequent carrier thermalization. Measurements were carried out in a range of temperature T = 10–300 K, with a calculated value *dn/dT* = 3.6 × 10^–5^ K^−1^ at T = 300 K.

Watanabe et al.^17^ investigated the temperature dependence of the refractive indices of 4H-SiC and GaN in a wavelength range from the near band edge (λ = 392 nm for 4H-SiC, λ = 367 nm for GaN) to infrared (λ = 1700 nm) from RT to T = 500 °C. Optical interference measurements were employed to precisely evaluate ordinary refractive indices. Near the band-edge region, the temperature dependence of the refractive index mainly originates from the temperature change of the bandgap. At λ = 450 nm, the TOCs of 4H-SiC and GaN were measured to be 7.8 × 10^–5^ K^−1^ and 1.6 × 10^–4^ K^−1^, respectively.

Xu et al.^18^ measured, by the method of minimum deviation, from T = 293 K to 493 K, the temperature-dependent refractive indices of 4H- and 6H-SiC over a spectral range from λ = 404.7 nm to λ = 2325.4 nm. The TOC dispersion formula as a function of wavelength and temperature was derived from the Sellmeier equation. For 4H-SiC, at the wavelength of λ = 450 nm, at T = 493 K, they calculated a *dn/dT* = 8.18 × 10^–5^ K^−1^, very close to what was reported in Ref.^17^. At the same temperature, for 6H-SiC, the TOC at the wavelength of λ = 1523 nm is 5.94 × 10^–5^ K^−1^, not far from the value of 5.54 × 10^–5^ K^−1^ reported in Ref.^11^.

To our knowledge, to date, there is however a lack of studies in the literature highlighting that the TOC for 4H-SiC and GaN, at λ = 1550 nm, is itself temperature-dependent, and in a not negligible manner. Therefore, in this letter, we report an experimental characterization of this coefficient for both wide bandgap materials, from RT up to T = 480 K, about, at said fiber optic communication wavelength. The results should be helpful for the proper design of 4H-SiC or GaN-based passive and active optoelectronic devices, like waveguides, couplers, interferometers, lasers, switches, modulators, etc. An unknown thermo-optic effect may cause, in fact, incorrect functioning of devices where the temperature dependence of the refractive index is itself temperature and wavelength dependent.

For example, small fluctuations of the TOC with the temperature can lead to significant yet undesirable shifts of resonances in microring-based devices, a phenomenon that is amplified in presence of unavoidable imperfections during the device fabrication processes that result in differences between the dimensions of waveguides, causing an imbalance in the spectral responses to temperature shifts.

## Experimental

**Figure 1:**
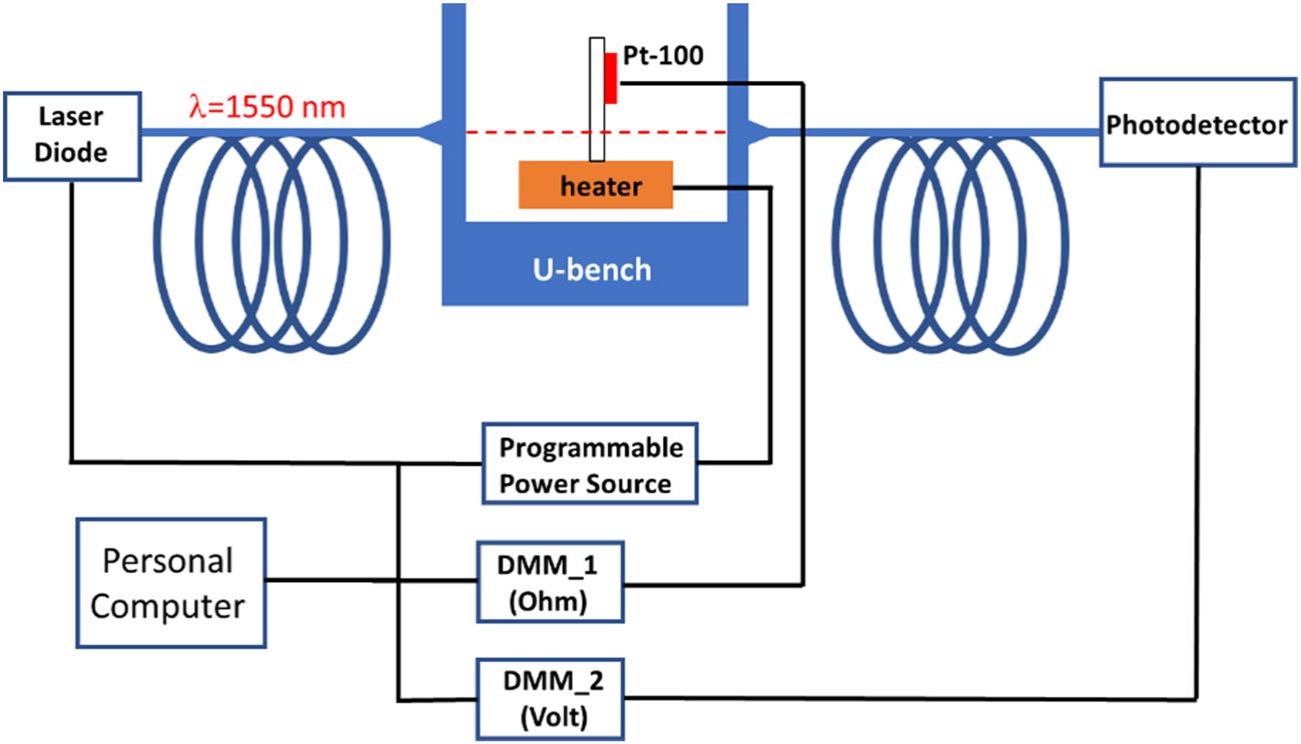
Experimental setup exploited for TOC characterization versus temperature.

The samples used in this work are two commercially available^19^ semi-insulating thick substrates of 4H-SiC and GaN, both < 001> oriented. The characterization of the thermo-optic coefficient was pursued with the experimental set-up schematically shown in Fig. 1.

In brief, the sample is contained in a U-bench (Thorlabs, FBC-1550-FC) and it is placed on a resistive heater to ensure uniform heating at the desired temperature. The actual temperature of the device under test (DUT) is monitored by a high accuracy PT-100 sensor, firmly glued on it close by the monochromatic light spot. A probe beam at the wavelength of λ = 1.55 μm, produced by a remotely controlled tunable laser diode, is launched across the sample, orthogonally to the surface. The sample, which is polished at an optical grade on both sides, behaves like a Fabry–Perot (FP) cavity. To calculate the value of *dn/dT*, the optical tuning and detuning of the FP cavity are monitored with the temperature change. The aim is to measure the temperature variation that is necessary for producing a complete FP detuning, by monitoring the transmitted radiation amplitude collected at the output by an InGaAs, switchable gain, amplified photodetector (Thorlabs, PDA10CS-EC).

For an FP cavity with symmetric mirrors, the transmitted light signal can be calculated by the following expression^20^:

1 $$I_{t} = \frac{{I_{o} }}{{1 + \frac{{4F^{2} }}{{\pi^{2} }}{\text{sin}}^{2} \emptyset }}$$

where *I*_*o*_ is the incident light intensity, *F* is the reflecting finesse of the cavity, and *ϕ* is the signal phase defined by $\emptyset = 2\pi nL/\lambda$, with *n* and *L* the refractive index and the length of the cavity, respectively.

The sin term confers periodicity to the equation, depending on the variation of the refractive index and on the FP cavity length, which both change with temperature. This trend is explicated by^21^:

2 $$\frac{\partial \phi }{{\partial T}} = \frac{2\pi L}{\lambda }\left( {\frac{\partial n}{{\partial T}} + \alpha \left( T \right)n\left( T \right)} \right)$$

where the term $\alpha = \partial L/L\partial T$ is the thermal expansion coefficient of the semiconducting material.

The temperature dependence of the thermal expansion coefficients for 4H-SiC and GaN are reported in Table 1 together with some specific geometrical parameters.

**Table 1:** Main features of the 4H-SiC and GaN samples.

	4H-SiC	GaN
Substrate^19^	Semi-insulating <0001>	Semi-insulating (Fe-doped) < 0001 >
Resistivity^19^	> 10^5^ Ω × cm	> 10^6^ Ω × cm
Roughness^19^	< 0.5 nm	< 0.5 nm
Energy gap (eV) (T = 300 K)	3.2	3.43
Thickness L (mm) ^19^	2	0.35
Thermal expansion coefficient *α* (10^–6^ K^−1^)	− 1.0971 × 10^−5^T^2^ + 1.8967 × 10^-2^ T-1.9755 ^20^	3.92 × 10^-3^ T + 2.42 ^23^
*n* (T = 300 K, λ = 1.55 μm)	2.56 ^22^	2.32 ^4,11,23^

## Results and experimental discussion

**Figure 2:**
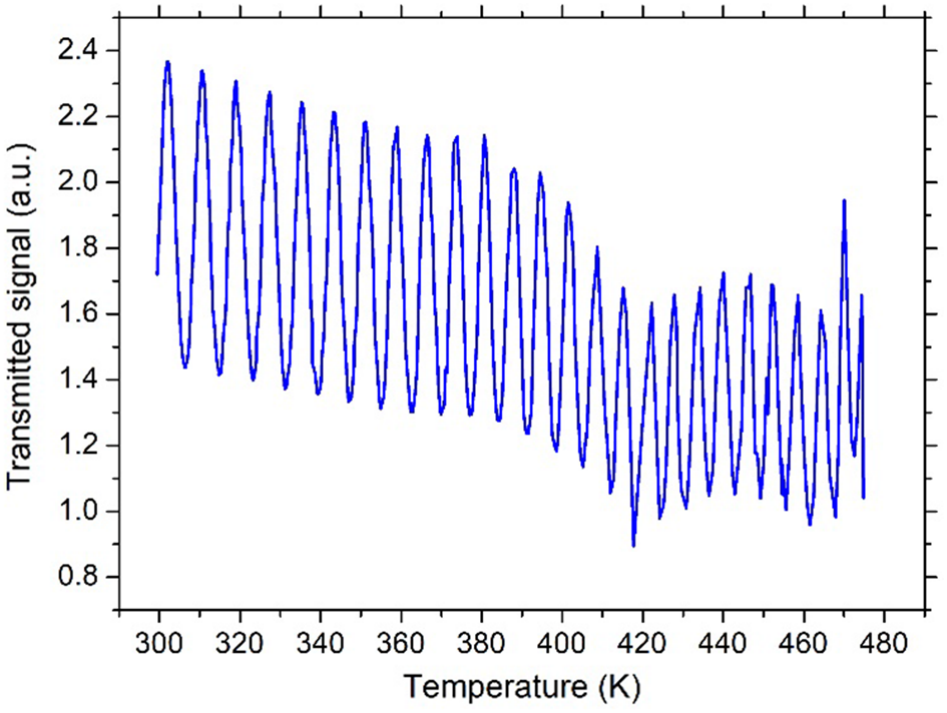
Example of a transmitted signal amplitude plot as a function of temperature for the 4H-SiC sample.

Several temperature sweeps were run on each sample. Figure 2 shows an example of the transmitted signal amplitude for the 4H-SiC substrate as a function of temperature from RT to T = 480 K.

**Figure 3:**
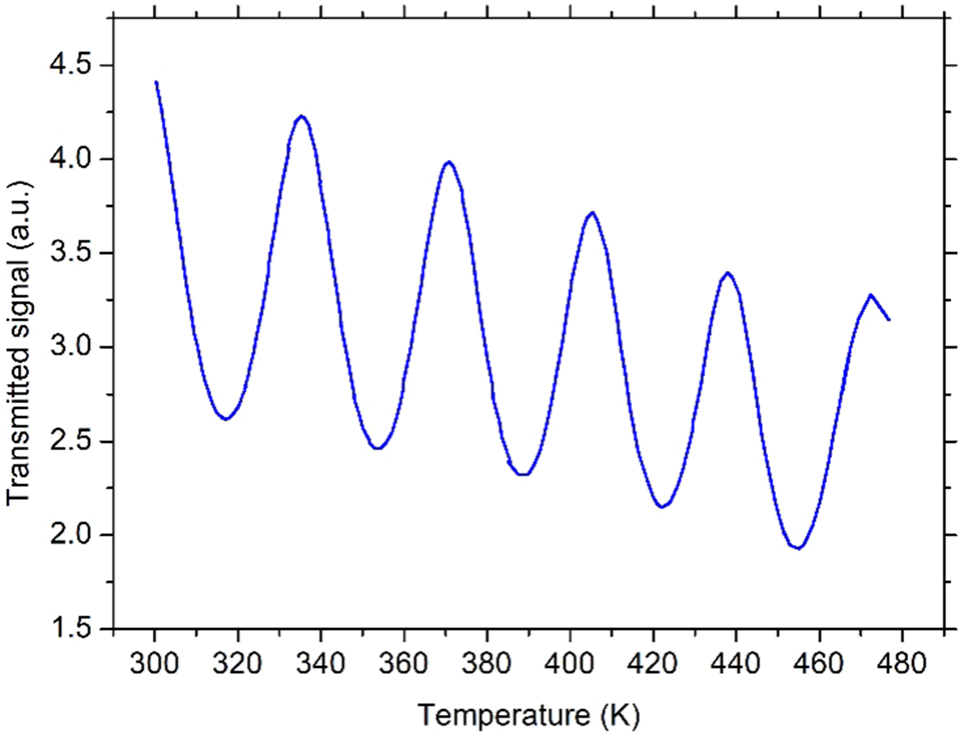
Example of a transmitted signal amplitude plot as a function of temperature for the GaN sample.

The same kind of plots was also obtained from the GaN sample. A sample graph is reported in Fig. 3 in the same range of temperature.

According to (2), the evaluation of the TOC, $dn/dT$, is obtained from the measurement of the distance in temperature between two consecutive transmission maxima (or minima), ${\Delta }T_{\pi }$, corresponding to a phase shift of the optical propagating field of $\Phi = \pi$.

Note that, at each temperature, *α(T)* is calculated according to the relevant equation in Table 1. In particular, the *α(T)* depence for GaN is the linear interpolation of the experimental data provided in Ref. 23, from 300 to 500 K. For what concerns *n(T)*, at each step its value is recursively updated with the value extracted at the previous temperature step.

It is worthwhile specifying that the amplitude drop present in Fig. 2 around 400 K is simply due to a sub-micrometric shift occurred in the mechanical assembly during the several-hours-long automated acquisition. Such events do not affect however the $dn/dT$ extraction, as it only depends on the distance in temperature between consecutive maxima or minima.

**Figure 4:**
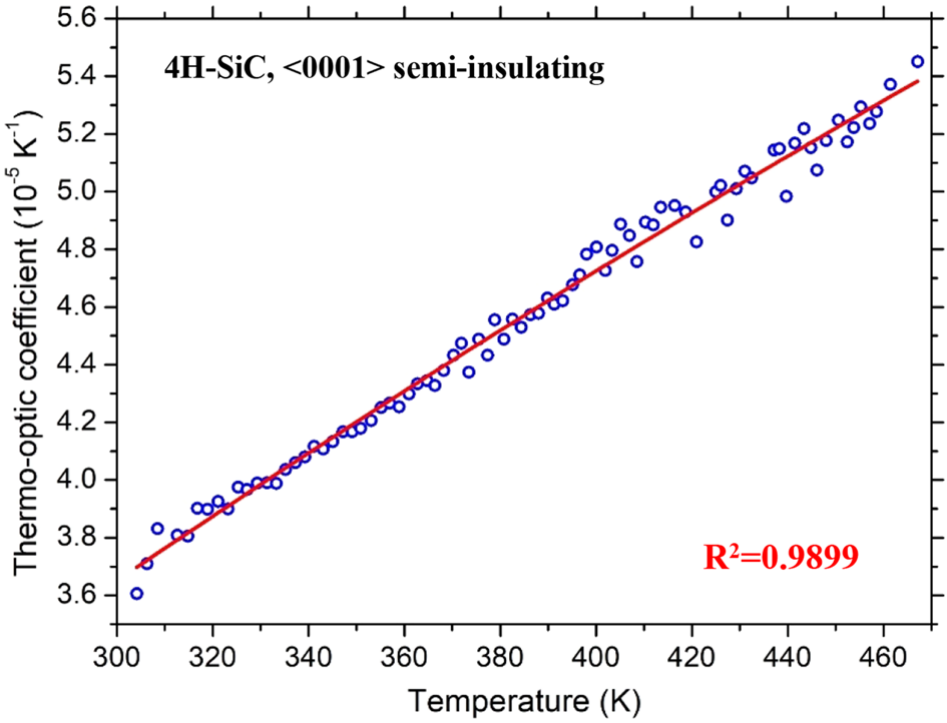
Thermo-optic coefficient as a function of temperature for the 4H-SiC sample.

Each DUT underwent several temperature ramps, from RT to T = 480 K and, again, from T = 480 K down to RT over a long time (one month). Each temperature sweep required approximately a full day of measurements. Once each operating temperature was reached, the system was kept in a stable condition for about ten minutes before starting the transmitted optical intensity measurement. Five successive and independent acquisitions of the photodetector output signal and the corresponding precise temperatures provided by the PT-100 sensor were subsequently averaged to get a single couple of measurement points, required, instead, only a few seconds. The overall results are summarized in Fig. 4 together with measurements made during the different measurement days.

**Figure 5:**
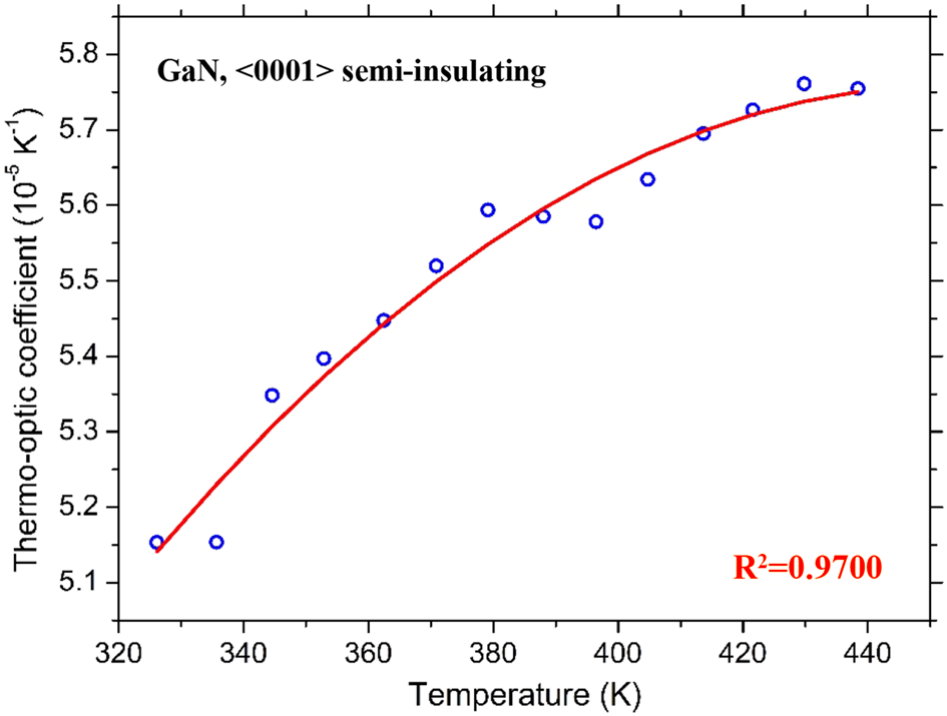
Thermo-optic coefficient as a function of temperature for the GaN sample.

The same experimental setup was exploited for the characterization of GaN substrate. All of the results are reported in Fig. 5. In this case, the distance in temperature between two consecutive transmission maxima (or minima) is larger with respect to the 4H-SiC substrate due to the reduced thickness of the FP cavity (L = 0.35 mm), therefore the number of the experimental points of TOC as a function of temperature is limited to only a few values. However, the dependence of the TOC on temperature variation is evident, although less remarkable if compared to the 4H-SiC.

Note that few comparable values of $dn/dT$ are found in the literature for 4H-SiC^17,18^ and GaN^17,23^, while no temperature dependence has been reported to date.

The experimental data obtained from measurements, at λ = 1550 nm, were modelled with the 2^nd^-order polynomial best-fits, *f*_*L*_*(T)*, described by the following equations:

3 $$\frac{\partial n}{{\partial T}} = - 5.55 \cdot 10^{ - 11} T^{2} + 1.46 \cdot 10^{ - 7} T - 2.36 \cdot 10^{ - 6} \quad for \, 4H - SiC$$

4 $$\frac{\partial n}{{\partial T}} = - 3.81 \cdot 10^{ - 10} T^{2} + 3.45 \cdot 10^{ - 7} T - 2.07 \cdot 10^{ - 5} \quad for\;GaN$$

The high coefficients of determination (*R*^2^), provided in Figs. 4 and 5, respectively 0.9899 and 0.9699 for 4H-SiC and GaN, demonstrate the good agreement between the experimental points and the polynomial fits. Note that the quadratic coefficient in (3) is very small, suggesting that the TOC dependence for 4H-SiC can be assumed, in fact, linear. Dropping the quadratic dependence leads to the more practical first-order approximation:

5 $$\frac{\partial n}{{\partial T}} = 1.03 \cdot 10^{ - 7} T - 5.77 \cdot 10^{ - 6} \quad for\;4H - SiC$$

at expenses of a negligible reduction of *R*^2^, which assumes in this case the value 0.9894.

**Figure 6:**
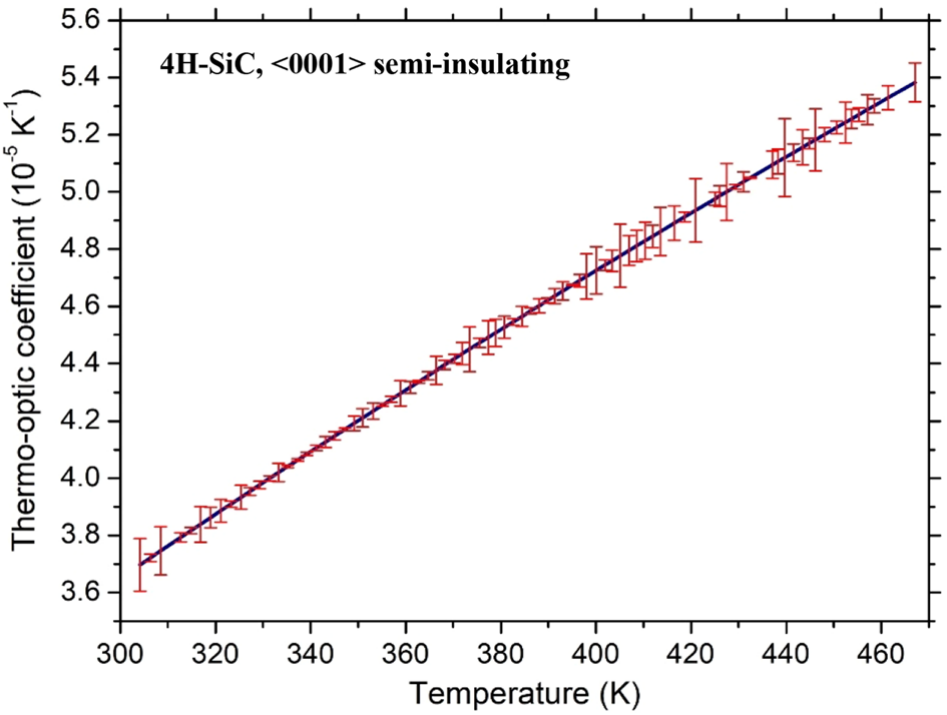
Polynomial fit and error bar of thermo-optic coefficient of 4H-SiC at the wavelength of 1.55 μm.

**Figure 7:**
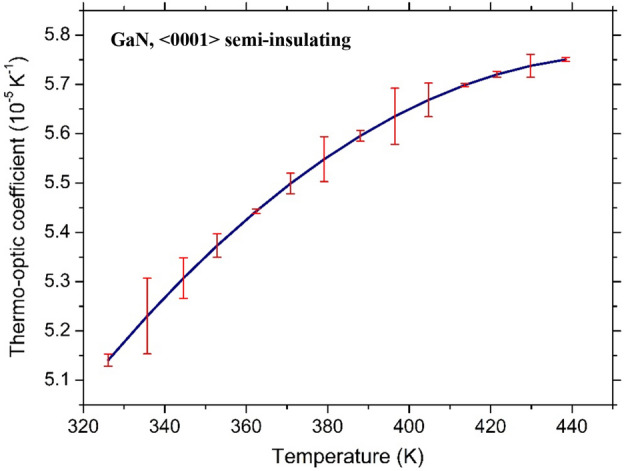
Polynomial fit and error bar of thermo-optic coefficient of GaN at the wavelength of 1.55 μm.

Another important parameter characterizing the goodness of the calculated TOCs, at the different considered temperatures during all the performed measurements (both positive and negative temperature ramps), with the polynomial best-fit, is the root-mean-square error (*rmse*). In Figs. 6 and 7 the polynomial fit and the relative error bars are reported for both substrates. The corresponding *rmse* values of 4H-SiC and GaN TOCs, in the investigated temperature ranges, are 4.74 · 10^–7^ K^−1^ and 3.78 · 10^–7^ K^−1^, respectively, about two orders of magnitude lower with respect to what can be calculated from Eqs. 3 and 4.

## Conclusions

In this study, the measurement of the thermo-optic coefficients ($dn/dT$) and their temperature dependences were reported on 4H-SiC and GaN. The results were accurately evaluated over a wide temperature range from RT to T = 480 K at the fiber-optic communication wavelength of λ = 1.55 μm. The experimental data were modelled with a 2nd-order polynomial best-fit and the coefficient of determination (*R*^2^) and the root mean square error (*rmse*) were calculated. The curves, fitting the TOCs achieved at different temperatures, matches very well the experimental data with a value of *R*^2^ of 0.9899 and 0.9699 for 4H-SiC and GaN, respectively. The TOC increases with temperature for both semiconductors, especially in 4H-SiC where it increases by about 25% for a 100 K temperature variation.

The results can be helpful for the proper design of SiC/GaN-based optoelectronic and nonlinear optical devices, operating in the infrared telecommunication region, that actively use, or are affected by, the refractive index change with temperature.
